# Engineered microRNA scaffolds for potent gene silencing in vivo

**DOI:** 10.1038/s41598-025-07061-y

**Published:** 2025-07-01

**Authors:** Giuseppe Militello, Alyssa Greig, Chongfeng Bi, Ana Vasileva, Maria I. Zavodszky, Shih-Ching Lo, Edward Guilmette, Pete Clarner, Bin Liu, Guruharsha Bhat, Junghae Suh, Lukas Dow, Johannes Zuber, Christof Fellmann, Prem K. Premsrirut

**Affiliations:** 1 Mirimus Inc., 760 Parkside Avenue, Suite 206, Brooklyn, NY 11226 USA; 2 Biogen Inc., 225 Binney St, Cambridge, MA 02142 USA; 3 Weill Cornell Medical College, 413 E 69th St (The Belfer Building), New York, NY 10021 USA; 4 Research Institute of Molecular Pathology (IMP), Campus-Vienna-Biocentre 1, Vienna, 1030 Austria; 5 CRISPR-X, CRISPR Therapeutics, 455 Mission Bay Boulevard South, San Francisco, CA 94158 USA; 6 SUNY Downstate Medical Center, 450 Clarkson Ave, Brooklyn, NY 11203 USA; 7 Department of Cellular and Molecular Pharmacology, University of California, San Francisco, San Francisco, CA 94158 USA; 8 Gladstone Institute of Data Science and Biotechnology, Gladstone Institutes, San Francisco, CA 94158 USA

**Keywords:** RNAi therapy, Molecular medicine, RNAi, Nucleic-acid therapeutics

## Abstract

RNA interference (RNAi) is emerging as a powerful strategy for therapeutic targeting of “undruggable” targets. However, efficacy of currently used siRNA-based therapies is often hindered by transient effects and limited modeling possibilities. Artificial microRNAs (amiRNAs or miRNA scaffolds) present a durable and precise approach to gene silencing, opening new avenues for developing long lasting targeted therapies. In this study, we engineered highly expressed primary miRNAs (pri-miRNAs) with sequence determinants known to enhance processing efficacy and precision. The resulting amiRNAs were extensively tested both in vitro and in vivo and proved to efficiently silence a target gene when virally delivered via adeno-associated virus (AAV) into mice brains. This study provides a set of novel amiRNAs with potential therapeutic application as well as a pipeline to generate and validate novel amiRNAs from endogenous pri-miRNAs.

**Supplementary Information:**

The online version contains supplementary material available at 10.1038/s41598-025-07061-y.

## Introduction

RNA interference (RNAi) is a ubiquitous biological process with enormous therapeutic potential. Short-interfering RNAs (siRNAs), short-hairpin RNAs (shRNAs), and artificial miRNAs (amiRNAs or miRNA scaffolds) are all RNAi effectors that engage the interference pathway at different levels and offer unique delivery and options for regulated expression^1^. AmiRNAs are delivered as gene cassettes expressed from an RNA Polymerase II promoter which allows for tissue- and cell type-specific control of gene silencing as well as long-lasting silencing compared to siRNAs and stem-loop shRNAs^2–4^. We and others have demonstrated how amiRNAs can efficiently silence any expressed transcript both in vitro and in vivo^2,5–7^ and several ongoing pre-clinical and clinical trials indicate the high potential of this class in a therapeutic setting^8,9^. However, current amiRNAs [e.g. miR-155, miR-30, miR-33, miR-451, miR-124, miR-101^3^] lack certain sequence determinants that are necessary to further enhance the silencing^10,11^. Here, we generated a set of novel amiRNAs by engineering ubiquitously and highly expressed primary miRNAs (pri-miRNAs) through the insertion of specific sequence determinants that are known to increase DROSHA and DICER processing^2,10,11^ and thus, enhancing both efficiency and precision compared to the previously reported miRE^2,12^ and miR-155^4,13,14^. The novel amiRNAs were tested and validated in vitro in both immortalized cell lines and human iPSC-derived neurons as well as in vivo through the delivery with rAAV9 to mice cortex to evaluate their efficacy in a pre-clinical setting.

## Results

### Design and screening of novel amiRNAs

**Fig. 1:**
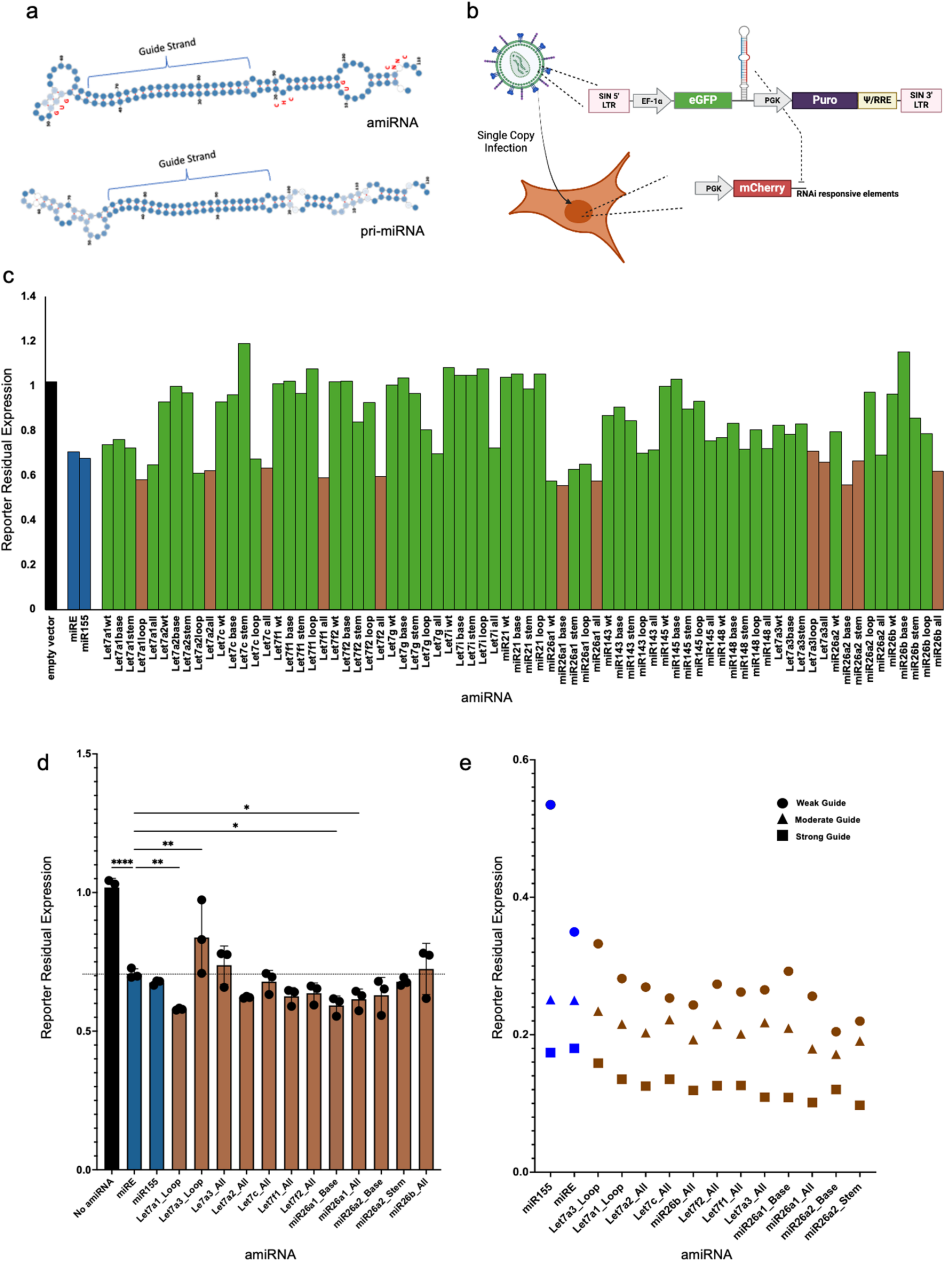
Design of novel amiRNAs and screening with reporter assay. (**a**) Secondary structure of the endogenous pri-Let7a1 (bottom) compared to the secondary structure of the engineered amiRNA derived from pre-Let7a1 with all the modifications indicated (top); sequence determinants are shown in red; darker blue indicates greater stability of the local structure. (**b**) A schematic of the reporter assay for the silencing efficiency evaluation of the novel amiRNAs; a reporter cell line stably expressing an mCherry cassette responsive to the screening guide (RNA responsive elements) was infected with a single copy of lentiviral particles encoding the amiRNAs under the control of the weak promoter EF-1α short. Single infection rate was confirmed by cytometric analysis three days post transduction (GFP + cells < 15% of total population). (**c**) Cytometry analysis six days post-transduction showing mCherry signal reduction was used as an indicator for silencing efficiency of the novel amiRNAs; an empty vector encodes for GFP only was used as a negative control. The mCherry signal from infected cells (GFP+) was normalized over the mCherry signal from non-infected cells (GFP−) within the same sample. Brown bars represent the amiRNAs selected for the rest of the studies described in (**d**). (**d**) Silencing efficiency of selected amiRNAs loaded with a weak guide sequence. (**e**) Reporter assay for novel amiRNAs loaded with guide sequences previously reported to have different efficiency ranging from weak, moderate to strong silencing; timepoints and readout used were the same as described for (**b**,**c**). Statistical analysis carried with one-way ANOVA; P values: *<0.05 - ** <0.01 - ***<0.001 - ****<0.0001.

 Our initial amiRNA designs stemmed from highly expressed, endogenous pri-miRNAs, rather than synthetic sequences, to avoid cytotoxicity or unpredictable effects when expressed at high copy number. We analyzed highly expressed human miRNAs from miRbase^15^ and chose the top 15 expressed miRNAs with a CNNC motif^2^ and supported by robust literature and experimental data, to serve as reference amiRNAs. To facilitate subcloning procedures, the corresponding pri-miRNA with a maximum length of 200nts was used as starting sequences (Supplemental Table 1). These sequences were subsequently modified in specific regions known to enhance DROSHA and DICER processing efficiency and precision^10,11,16,17^ (Fig. 1a, Supplemental Table 2): (a) Base of the stem modification: GU dinucleotide in position − 13 and − 14 from DROSHA putative 5′ cleaving site, unstructured base of the stem, and 35 bp long stem. (b) CHC insertion: addition of the CHC bulge in position − 6 from DROSHA putative 5′ cleaving site and removal of all bulges between positions 0 and − 13. (c) Loop modification: removal of the endogenous loop and addition of miR-30a loop. (d) all modifications combined. For experimental screening purposes, the guide/passenger duplex was replaced with a sequence targeting the 3′UTR of a fluorescent gene expressed by a U251-based reporter cell line. To better discriminate between amiRNA efficiencies and to avoid saturation of the silencing, a guide/passenger duplex known to yield moderate knockdown^5^ was embedded and the expression of the amiRNAs was driven by the weak promoter EF-1α short (Fig. 1b). Following lentiviral packaging, the reporter line was infected at single copy efficiency^5,6^ and the residual fluorescence was measured to assess the knockdown efficiency; we observed that several amiRNAs achieved silencing efficiencies equivalent to or exceeding miRE and miR-155 by up to 15% in our first biological replicate (Fig. 1c). Following the removal of non-performing amiRNAs, we repeated the experiment for two more biological replicates using 12 varied candidates (Fig. 1d) and confirmed their superior silencing using a range of guides with differing potencies (Fig. 1e).

### Validation of the novel amiRNAs

**Fig. 2:**
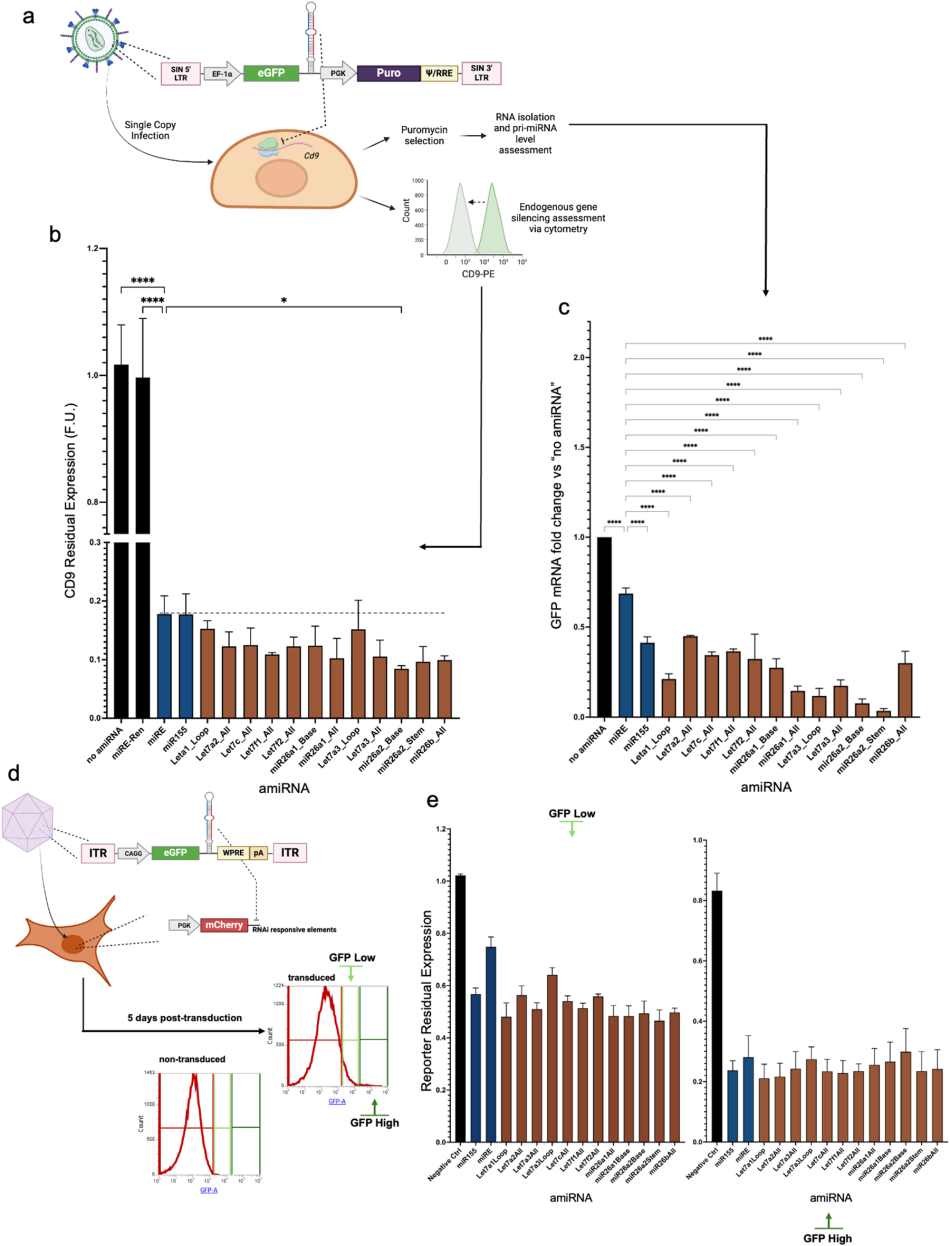
Validation of the novel amiRNAs for the silencing of endogenous genes and delivery with rAAV9 vectors. (**a**) Schematic of single-copy lentiviral infection for silencing *Cd9* in 3T3 cells. The single infection rate was confirmed by cytometric analysis three days post-transduction, with GFP + cells comprising less than 15% of the total population. (**b**) Six days post-transduction, a fraction of the cells was stained for CD9 and analyzed by cytometry to quantify the knockdown efficiency. (**c**) The remaining fraction was selected with puromycin to obtain a homogeneous pool of cells expressing the amiRNAs from a single locus, and RNAs were isolated to quantify the residual amount of GFP as a proxy for processing efficiency. (**d**) Schematic of rAAV9 infection in the reporter line. (**e**) Five days post-transduction, cytometry was employed to assess mCherry silencing in GFP-high (high viral cargo expression) and GFP-low (low viral cargo expression) populations, with mCherry expression normalized to that in GFP- (non-infected) cells within the same sample. “Negative control” indicates Let7f2 WT, a non-functional amiRNA from the screening shown in Fig. 1b,c. Statistical analysis was performed using one-way ANOVA, with P-values indicated as *<0.05, **<0.01, ***<0.001, and ****<0.0001.

 To validate the knockdown efficiency of the candidates, we performed experiments targeting an endogenous transcript. A guide targeting the surface marker CD9 was incorporated into the amiRNAs, and 3T3 cells were infected with a single copy of the lentiviral vector (Fig. 2a). Six days post-infection, cells were analyzed by flow cytometry, which revealed a 14–52% increase in silencing efficiency of the novel amiRNAs compared to miRE and miR-155 (Fig. 2b). In addition, the infected cells were selected to obtain a population expressing the amiRNAs from a single copy. Following RNA isolation, we quantified the residual amount of GFP mRNA as proxy for amiRNA processing^2^ and demonstrated that eleven out of twelve amiRNAs had lower levels of GFP transcript (Fig. 2c), confirming enhanced processing which supports the increased silencing activity observed. Next, we tested the functionality of the novel amiRNAs when delivered by recombinant adeno-associated virus 9 (rAAV9), a widely used vector for gene therapy purposes^18–21^. The U251-based mCherry reporter line was transduced with rAAV9 encoding the amiRNAs at high MOI and subjected to cytometry analyses, which showed the ability of the amiRNAs to induce silencing of the fluorescent target in cells that maintained high expression of the viral cargo five days post-transduction (Fig. 2d,e).

### AmiRNAs processing studies

**Fig. 3:**
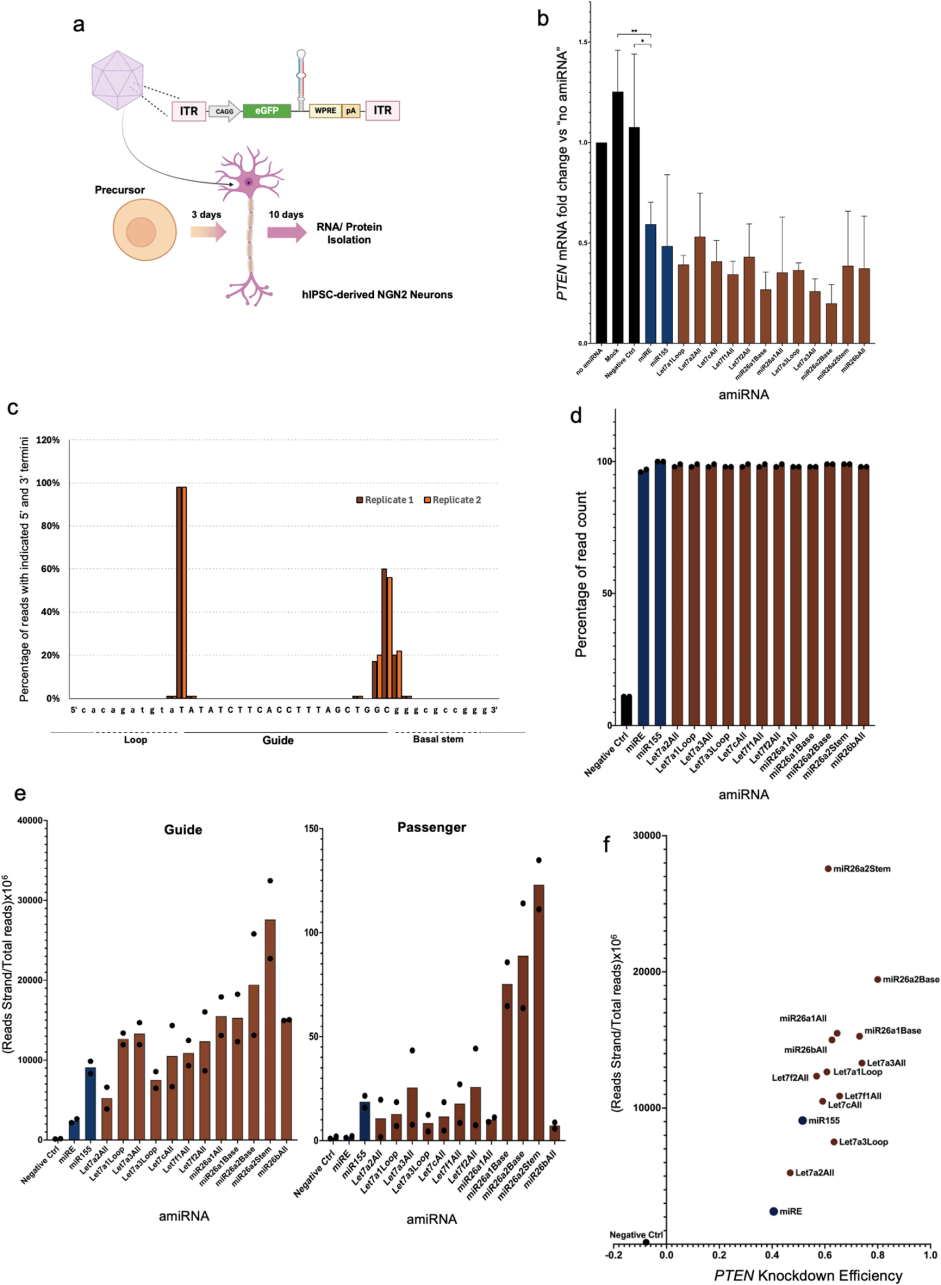
AmiRNAs processing studies in hIPSC-derived neurons. (**a**) Schematic of transduction of hIPSC-derived NGN2 neurons with rAAV9 encoding the novel amiRNAs loaded with a guide against *PTEN*. (**b**) qPCR was used to quantify the residual expression of *PTEN*; “no amiRNA” refers to the virus encoding GFP only. “Negative control” indicates Let7f2 WT, a non-functional amiRNA from the screening in Fig. 1b,c; “Mock” refers to non-transduced cells. (**c**,**d**) Analysis of mature amiRNA processing precision: (**c**) The percentage of reads starting and ending with the corresponding nucleotide. miR26bAll is used as an example; the guide strand sequence is in uppercase, and the adjacent amiRNA nucleotides are in lowercase. (**d**) The percentage of reads starting with the correct 5′ nucleotide for each amiRNA; dots represent replicates. (**e**) Normalized quantification of guide and passenger strands. (**f**) Correlation [Pearson r value: 0.6141, P-value (two-tailed): 0.0195] between guide strand amount (determined from small RNA sequencing) and silencing efficiency (determined from qPCR; “1” = 100% silencing). Statistical analysis was carried out using one-way ANOVA and Pearson’s Correlation test. P-values: *<0.05, **<0.01, ***<0.001, ****<0.0001.

 An important characteristic of our amiRNA designs is not only their processing efficiency by the RNAi maturation machinery, but also the accuracy of processing whereby a homogenous pool of guide strands with identical seed sequence is generated. This precise processing decreases the chance of off-targeting and secures the biased loading of the guide strand onto RISC, which further enhances the overall silencing efficiency^22^. Human iPSC-derived NGN2 neurons were selected as our experimental model because of their direct relevance to therapeutic applications and their ability to more closely mimic the biological conditions found in patients^23^. As depicted in Fig. 3a, rAAV9 encoding the novel amiRNAs loaded with a guide strand against the endogenous *PTEN* was used for transduction and gene silencing of the target gene was confirmed (Fig. 3b). We also isolated total RNA ten days post-transduction and performed small-RNAseq analyses to study the pool of guide and passenger strand being produced from the viral vectors. In every case, the fraction of guide strands starting with the expected 5’ nucleotide was greater than 98%, indicating precise processing of the amiRNAs (Fig. 3c,d) to form a homogenous pool with identical seed sequence. Next, we quantified guide and passenger strands and confirmed the guide was 10^2^–10^3^-fold more abundant than the latter (Fig. 3e, S1B). Importantly, we also observed that the number of guides being produced by many of the novel amiRNAs was far greater than miRE and miR-155 (Fig. 3e) and all the endogenous miRNAs (Figure S1B, Supplemental Table 4), with the magnitude of silencing being correlated to the amount of guide being produced by each amiRNA (Fig. 3f). Moreover, the expression of the amiRNAs did not cause dramatic changes in the expression of the endogenous miRNAs (Fig. S2A); similarly, bulk-RNAseq analysis revealed that, with exclusion of the scaffold Let7f2All, the global transcriptome was not severely impacted by the expression of the amiRNAs (Fig. S3A). Taken together, the results demonstrate the importance of precise and efficient amiRNA processing to improve silencing strength and precision with minimal impact on the cellular miRome and transcriptome.

**Fig. 4:**
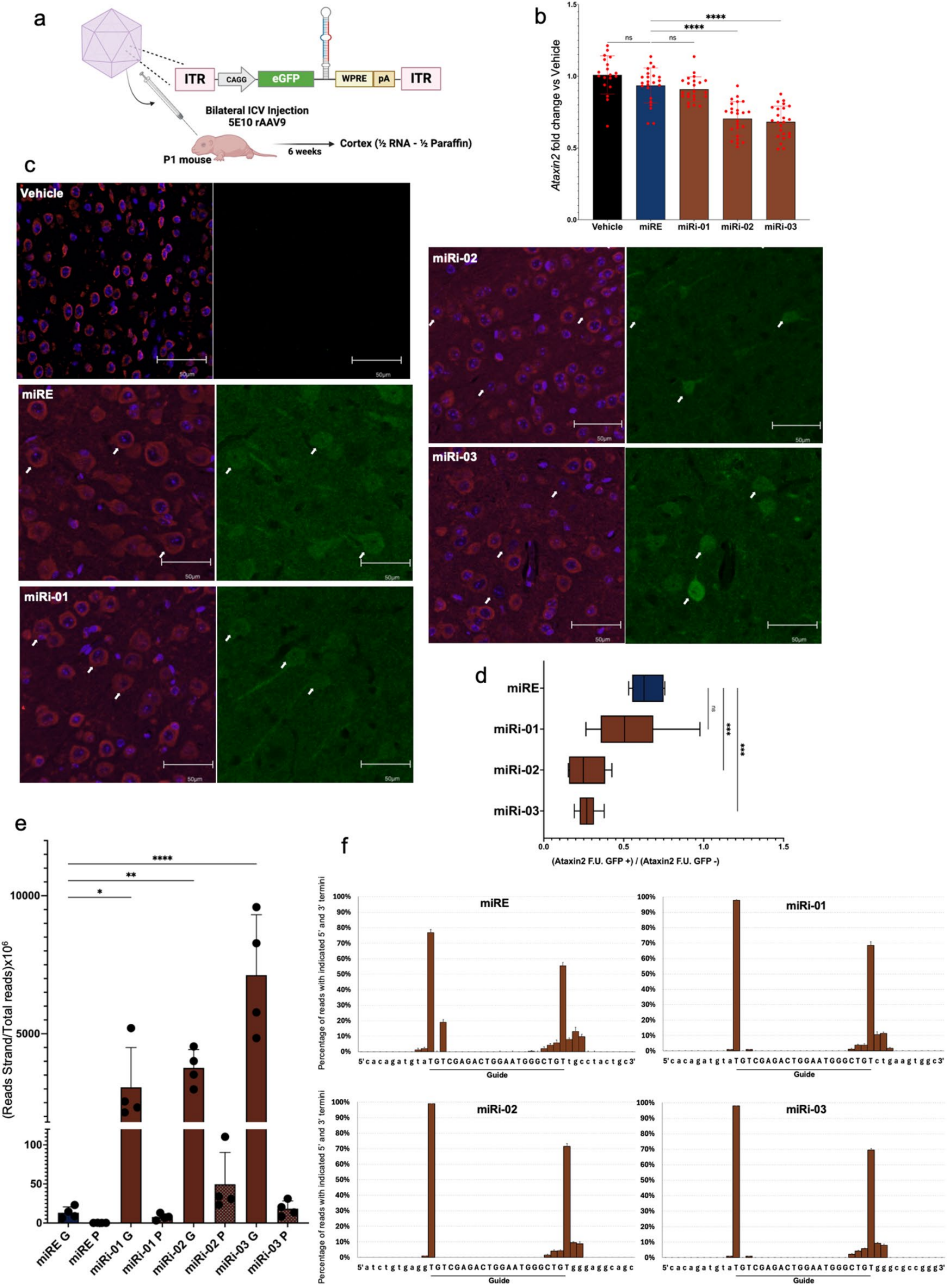
Delivery of novel amiRNAs in vivo via Intracerebroventricular injection of rAAV9. (**a**) Schematic of in vivo intracerebroventricular injection of rAAV9 encoding the novel amiRNAs loaded with a guide targeting *Ataxin2*. (**b**) qPCR analysis to measure *Ataxin2* expression; “Vehicle” refers to PBS treatment. (**c**) Representative immunofluorescence images showing ATAXIN2 (red), GFP (green), and nuclei (blue). (**d**) Quantification of ATAXIN2 expression in GFP-positive (transduced) versus GFP-negative (non-transduced) cells (*n* = 4). (**e**) Normalized quantification of guide (G) and passenger (P) strands. (**f**) Analysis of mature amiRNA processing precision: the percentage of reads starting and ending with the correct nucleotide; the guide strand sequence is in uppercase, and adjacent amiRNA nucleotides are in lowercase. Statistical analysis was performed using one-way ANOVA. P-values: *<0.05, **<0.01, ***<0.001, ****<0.0001.

In vivo validation. To confirm the results obtained in vitro could be translated in vivo, we further tested a subset of novel amiRNAs through the delivery with rAAV9 into mice. We chose three amiRNAs with different silencing efficacy (Fig. 3f): Let7a3_Loop (or miRi-01), miR26a2_Base (or miRi-02), miR26b_All (or miRi-03). The known miRE was added for comparison, and the amiRNAs were engineered with a guide/passenger strand targeting the gene *Ataxin-2.* rAAV9 vectors encoding the amiRNAs were administered in day 1 postnatal mice brain via intracerebroventricular (ICV) injection at the titer of 5E10 viral genome copies. Six-weeks post injection, mice were sacrificed for endpoint studies (Fig. 4a). Total RNA isolated from cortexes showed minimal silencing of *Ataxin2* when using the miRE and the suboptimal miRi01 amiRNAs; in contrast, miRi02 and miRi03 yielded enhanced silencing, in line with our in vitro observations (Fig. 4b). To complement the bulk RNA analyses and estimate cell-level target knockdown, we performed immunofluorescence analyses in PFA-embedded sections from the same cortexes and measured ATAXIN2 expression in GFP + cells (transduced) and GFP- cells (non-transduced) to more precisely quantify the silencing. As shown in Fig. 4c,d, we confirmed that the novel amiRNAs outperform miRE and yield improved silencing when expressed in vivo. To confirm the efficient processing of our amiRNAs in vivo, similar to the in vitro studies, small RNA-seq analysis showed a 10^2^–10^3^-fold greater production of the guide strand compared to the negligible presence of the passenger strand (Fig. 4e, S1D). Moreover, we could confirm the precise processing and homogenous pool of guide strand (Fig. 4f) as well as the high abundance of the mature amiRNA guide strand compared to the endogenous miRNAs pool (Fig. S1C, Supplemental Table 5). No dramatic impacts on miRome (Fig. S2B) and transcriptome (Fig. S3B) were detected, with the few differentially expressed genes showing no seed-match with the guide strand of the amiRNAs; however, it is important to note that the in vivo infection efficiency was not complete; therefore, this observation, as well as the quantification of mature amiRNA and *Ataxin2* silencing at the RNA level, may be underestimated due to the contribution of non-infected cells in the bulk sample. Lastly, to rigorously evaluate potential confounding factors related to vector quality, we performed a comprehensive assessment of rAAV9 genome integrity using two-dimensional droplet digital polymerase chain reaction (2D-ddPCR). The data presented in Fig. S4 unequivocally demonstrate that all rAAV9 preparations exhibited comparable genome integrity profiles. Consequently, we can confidently assert that the observed variations in the silencing are not attributable to significant differences in the physical integrity of the delivered viral genomes.

## Discussion

RNAi therapeutics are increasingly gaining momentum for the treatment of diseases in which genome editing and/or molecularly targeted therapies are not employable^1,24^. Most of the current pre-clinical and clinical trials are based on siRNA therapeutics that, while powerful, show limitations such as poor endosomal escape, limited half-life thus requiring multiple dose regimens, and difficulty in restraining silencing to specific tissues^25^. AmiRNAs can overcome these limitations^3^. They can be delivered with viral vectors to achieve long-term gene silencing following a single dose (e.g., clinical trial for the treatment of Huntington disease NCT0543017-NCT04120493 and Sickle Cells Disease NCT03282656 - NCT05353647). Furthermore, extensive research in academia and industry indicates that viral capsid engineering is a promising approach for achieving tissue-specific delivery^18,26–29^. Importantly, amiRNA expression is driven by RNA polymerase II promoters which enables the use of tissue specific promoters that can further restrict gene silencing to the cells/tissue of interest and thus limit off-target effects observed with the systemic delivery of siRNAs.

In this study, we have not only generated a new set of amiRNAs, but we have also outlined a design and experimental pipeline to generate amiRNAs from potentially any endogenous pri-miRNA. Such a design strategy, however, was not successful in certain instances (Fig. 1B), indicating the existence of unknown processing regulators as well the fact that certain pri- and pre-miRNAs may not need the presence of the currently known sequence and structure determinants^11^ for efficient and precise processing.

It is important to note that due to differences in primary and secondary structure, amiRNAs can have different stability/efficiency in different tissues and interestingly, a study showed how pri-miRNAs (and thus amiRNAs) can be processed in a tissue-specific manner^30^. This opens a fascinating venue in which amiRNAs that are processed only in certain tissues of interest can further improve the specificity through a “three-steps” control of the silencing: delivery, expression, and processing.

In summary, our novel amiRNAs and experimental pipeline has the potential to enrich the RNAi toolkit available to the scientific community and opens new paths for the generation of safer and more powerful RNAi therapeutics.

## Methods

### Cloning of vectors and generation of viral particles

The retroviral vectors used to generate the reporter lines were based on the pMSCV backbone and were modified to express mCherry, along with RNAi-responsive elements in the 3′UTR and a Blasticidin resistance cassette. Retroviral particles were produced by transfecting the helper-free Phoenix-AMPHO producer cell line (ATCC) with the retroviral plasmid using Lipofectamine 2000 (Thermo Fisher Scientific) according to the manufacturer’s instructions. Two days after transfection, the viral-containing supernatant was collected and filtered through 0.45 µM PVDF syringe filters. The filtered supernatant was then diluted 1:200 and used to infect naïve U251 cells to generate the U251-based reporter line. Once it was confirmed that the reporter gene was expressed from a single copy (with fewer than 15% of cells expressing mCherry 2 days post-transduction), the cells were selected with 5 ng/µL Blasticidin. Clones were then derived, and the clone showing the most uniform mCherry expression was selected for further experiments.

For the expression of amiRNAs, the cargo lentiviral plasmid was created by replacing the SFFV promoter in the pRRL-based SGEP^2^ plasmid with the EF-1α short promoter. Each amiRNA (listed in Supplemental Table 1) was ordered as an Ultramer oligo (IDT) and cloned into the cargo plasmid using the NEB Gibson Assembly MasterMix Kit, following the manufacturer’s protocol. Lentiviral particles were produced by transfecting 70% confluent Lenti-X 293T cells (Takara Bio) with Lipofectamine 2000 (Thermo Fisher Scientific) according to the manufacturer’s instructions. The plasmid amounts used were 50% for the lentiviral cargo plasmid, 37.5% for the p-CMV-dR8.2 helper plasmid, and 12.5% for the p-CMV-VSVG plasmid, relative to the total plasmid mass. Two days post-transfection, the supernatant containing the lentiviruses was collected, filtered through 0.45 µM PVDF syringe filters, and diluted 1:100–1:200 before infecting the U251 reporter line.

Plasmid maps of all the vectors used in this study are available upon request. 

### Reporter assay and CD9 cytometry analysis

The reporter assay was conducted by transducing the U251-based reporter line with diluted (1:100–200) lentiviral vectors encoding the amiRNAs at a single copy. Three days post-transduction, an aliquot of cells was analyzed by flow cytometry to ensure that less than 15% of the total cells expressed GFP (GFP + cells indicate single-copy integrations). Six days post-transduction, the residual mCherry expression was quantified using the formula: mCherry Χ-Median (GFP+)/mCherry Χ-Median (GFP−), with analysis performed on an Attune NxT Cytometer (Thermo Fisher Scientific).

For CD9 staining, NIH/3T3 cells were transduced with diluted (1:100–200) lentiviral preparations encoding the amiRNAs at a single copy. Six days after transduction, 500,000 cells were stained with 0.5 µg of CD9 PE-conjugated Monoclonal Antibody (eBioKMC8 (KMC8)—eBioscience™), diluted in Flow Cytometry Staining Buffer (eBioscience™). After a 30-minutes incubation at + 4 °C in the dark, the cells were washed twice with the staining buffer and analyzed by flow cytometry. Rat IgG2a kappa Isotype Control (eBR2a), PE (eBioscience™), was used as a negative control (data not shown).

### Cell culture

U251 cells were purchased from AddexBio and cultured in Eagle’s Minimum Essential Medium (EMEM) supplemented with 10% fetal bovine serum (FBS) and 1× Penicillin-Streptomycin. Phoenix AMPHO, NIH/3T3 (purchased from ATCC), and Lenti-X 293T (purchased from TakaraBio) cells were cultured in Dulbecco’s Modified Eagle Medium (DMEM), also supplemented with 10% FBS and 1× Penicillin-Streptomycin.

Partially differentiated human induced pluripotent stem cell (hiPSC)-derived Neurogenin 2 (NGN2) neurons were provided by the Biogen Stem Cell Biology group. A total of 500,000 cells were plated onto poly-D-lysine-coated 6-well plates in a differentiation medium composed of a 1:1 mixture of DMEM-F12 and Neurobasal media, supplemented with 1× Penicillin-Streptomycin, 1× GlutaMAX, 0.5× N2 Supplement, 0.5× B27 Supplement, 10 mg/mL Doxycycline, 200 mM ascorbic acid, 1 mM dibutyryl cyclic AMP (dbcAMP), 1× CultureOne Supplement, 10 ng/mL brain-derived neurotrophic factor (BDNF), and 10 ng/mL glial-derived neurotrophic factor (GDNF). Seven days post-thawing, the medium was replaced with a feeding medium containing all the above components except for the CultureOne Supplement and Doxycycline.

Recombinant adeno-associated virus serotype 9 (rAAV9) was produced and titrated (ddPCR titration) by PackGene Biotech Inc. (Houston, Texas, USA). Transduction of NGN2 neurons was performed three days post-thawing by directly adding the viral preparation to the medium at a multiplicity of infection (MOI) of 450,000 viral genome copies per cell.

### Total RNA isolation, qRT-PCR analysis, and small RNA sequencing

Total RNA was isolated from NIH/3T3 cells, human iPSC-derived NGN2 neurons (at least 500,000 cells per sample), and flash-frozen mouse cortices using TRIzol reagent (Thermo Fisher Scientific) according to the manufacturer’s protocol. RNA concentrations were quantified using a NanoDrop Lite Spectrophotometer (Thermo Fisher Scientific). For the mouse cortex samples, the tissue was homogenized in TRIzol using a disposable pestle in 1.5 mL tubes. A total of 250 ng of purified RNA was used for cDNA synthesis using the Maxima H Minus cDNA Synthesis Master Mix with dsDNase (Thermo Fisher Scientific). Quantitative PCR (qPCR) was performed with gene-specific primers (listed in Supplemental Table 3) and SYBR Green PCR Master Mix (Thermo Fisher Scientific) on the QuantStudio 7 Pro Real-Time PCR System (Thermo Fisher Scientific).

For small RNA sequencing (small-RNAseq) studies, 2 µg of total RNA per sample was sent to Novogene Corporation Inc. (Sacramento, California, USA) for quality control, library construction, and next-generation sequencing (NovaSeq, SE50, Illumina). Briefly, the data were analyzed as follows:

Quality Control: FastQC (http://www.bioinformatics.babraham.ac.uk/projects/fastqc/) was employed to evaluate the quality of the raw sequence data, ensuring that data integrity and base quality were within acceptable ranges. Fastp software^31^ was used for adapter removal and quality trimming. For the in vivo studies, Kraken2^32^ was utilized to eliminate contaminations from microbial and human cells. A second round of quality assessment was performed using FastQC to verify that the trimmed data met quality standards and contained minimal microbial and human cell contamination.

Preparation of Reference Vector Genomes: Reference genomes were prepared with an additional 10 bases on each side of the guide and passenger for each artificial miRNA. This preparation facilitated precise alignment and quantification of miRNA reads. Alignments were conducted against the prepared vector genomes individually using BWA software to prevent cross-mapping issues^33^. The alignment process involved sorting the SAM files using BWA commands bwa aln and bwa samse, followed by conversion to BAM files using SAMtools^34^. Custom in-house scripts (available on request) were developed to quantify the counts of aligned reads for the guide and passenger miRNAs. Analysis was performed using SAMtools and custom Python scripts to determine read alignment accuracy and integrity based on CIGAR Score and Flags. This quantification was based on following criteria: (a) Full-Length Match: Reads were required to match a minimum length of 18 and a maximum length of 24 nucleotides to ensure accurate miRNA representation. (b) No Insertions or Deletions: Only matches (e.g., 22 M) were considered valid to maintain consistency in alignment analysis.

Precision Processing Analysis and Visualization: quantification data was analyzed by concatenating the 22 nucleotide reads with 10 bases from the left and right flanking regions, resulting in a total of 42 DNA bases. This approach enabled detailed examination of miRNA processing precision either from the 5’ end or 3’ end. The counts of each starting and ending mapping site were calculated as percentages, facilitating subsequent data visualization processes for the precision processing analysis.

### Vertebrate animals housing and handling

Six weeks old C57BL/6 mice were purchased from The Charles River Laboratories and were maintained in the animal facility of the SUNY Downstate Medical Center. The research conducted in this study complies with all relevant ethical regulations. All procedures involving mice and experimental protocols were approved by the Institutional Animal Care and Use Committee of the SUNY Downstate Medical Center (IACUC protocol number: 23-10633) and data are reported according to ARRIVE guidelines.

### Intracranial cerebroventricular injection of rAAV9

Newborn C57BL/6 mice (P1) were anesthetized using isoflurane. A Hamilton Neuros Syringe with a 33-gauge needle was used to deliver 5 µL of viral solution (bilateral injection; 2.5 µL/hemisphere), containing 5E10 viral genome copies, into the brain at a lateral position of approximately 0.8–1 mm from the sagittal suture, midway between the lambda and bregma landmarks. 0.1% Fast Green FCF dye was added to the solution to visually confirm the spread of the injection into the ventricles. Twenty to thirty pups per cohort were treated in the study by an operator who was blinded to the treatment. After injection, pups were monitored for recovery and returned to their mothers. Six weeks post-injection, mice were euthanized. One hemisphere of the brain was flash-frozen for RNA extraction, while the other hemisphere was fixed in 10% Neutral Buffered Formalin, followed by storage in 70% ethanol and paraffin embedding for histological analysis.

### Immunostaining and quantification of ATAXIN2 signal in cortexes

Fixed brain tissue was sent to HistoWiz, Inc. (Long Island City, New York, USA) for processing, including embedding, staining, and imaging. The tissues were stained with antibodies against ATAXIN2 (Proteintech, 21776-1-AP) and GFP (Thermo Fisher Scientific, A-11122). Immunofluorescence images were analyzed using ImageJ software to quantify ATAXIN2 expression in both transduced (GFP+) and non-transduced (GFP−) cells. Background noise was removed from the images, and the GFP signal was used to identify transduced cells. In the case of non-transduced cells, ImageJ’s “Analyze Particles” function was used to select all cells. For both cell groups, the integrated density of the ATAXIN2 immunofluorescence signal was determined for each selected cell using ImageJ’s “Analyze -> Measure” function, and the “Mean Gray Value” was recorded.

### Genome integrity analysis by 2D-ddPCR

rAAV9 samples were treated with DNase I for 30 min at 37 °C. A capsid lysis buffer was then added to the samples, and they were heat treated to release encapsidated genomes. Dilutions of extracted genomes were prepared and combined with a mixture of ddPCR Supermix for Probes (no dUTP), a custom primer/probe for the CMV enhancer (VIC labeled probe), and a custom primer/probe for the hGH polyA (FAM labeled probe). 2D-ddPCR was performed by following the ddPCR workflow according to the manufacturer’s instructions using the AutoDG Droplet Generator and QX200 Reader. Percent of intact genomes for each sample were determined by calculating the percent of ddPCR droplets that were PCR positive for both the CMV enhancer and hGH polyA within the same droplet.

### Statistical analysis

Statistical analyses were performed using GraphPad Prism version 10.4.0 (527). Statistical significance was determined using the tests specified in the respective figure legends. Results are shown as mean ± SD.

## Electronic supplementary material

Below is the link to the electronic supplementary material.

Supplementary Material 1

Supplementary Material 2

Supplementary Material 3

Supplementary Material 4

Supplementary Material 5

Supplementary Material 6

Supplementary Material 7

Supplementary Material 8

Supplementary Material 9

Supplementary Material 10

## Data Availability

The datasets generated during the current study are available in the Sequence Read Archive (SRA) repository, accession numbers: PRJNA1204069—http://www.ncbi.nlm.nih.gov/bioproject/1204069 (in vivo) and PRJNA1204072—http://www.ncbi.nlm.nih.gov/bioproject/1204072 (in vitro). Alternatively, please contact the corresponding author, Giuseppe Militello (militello@mirimus.com) to obtain the research data.
