# Supplementary figures and images for: Engineered microRNA scaffolds for potent gene silencing in vivo
Source: Sci Rep. 2025 Jul 1;15:21419. doi: 10.1038/s41598-025-07061-y (PMC12218229; doi:10.1038/s41598-025-07061-y)

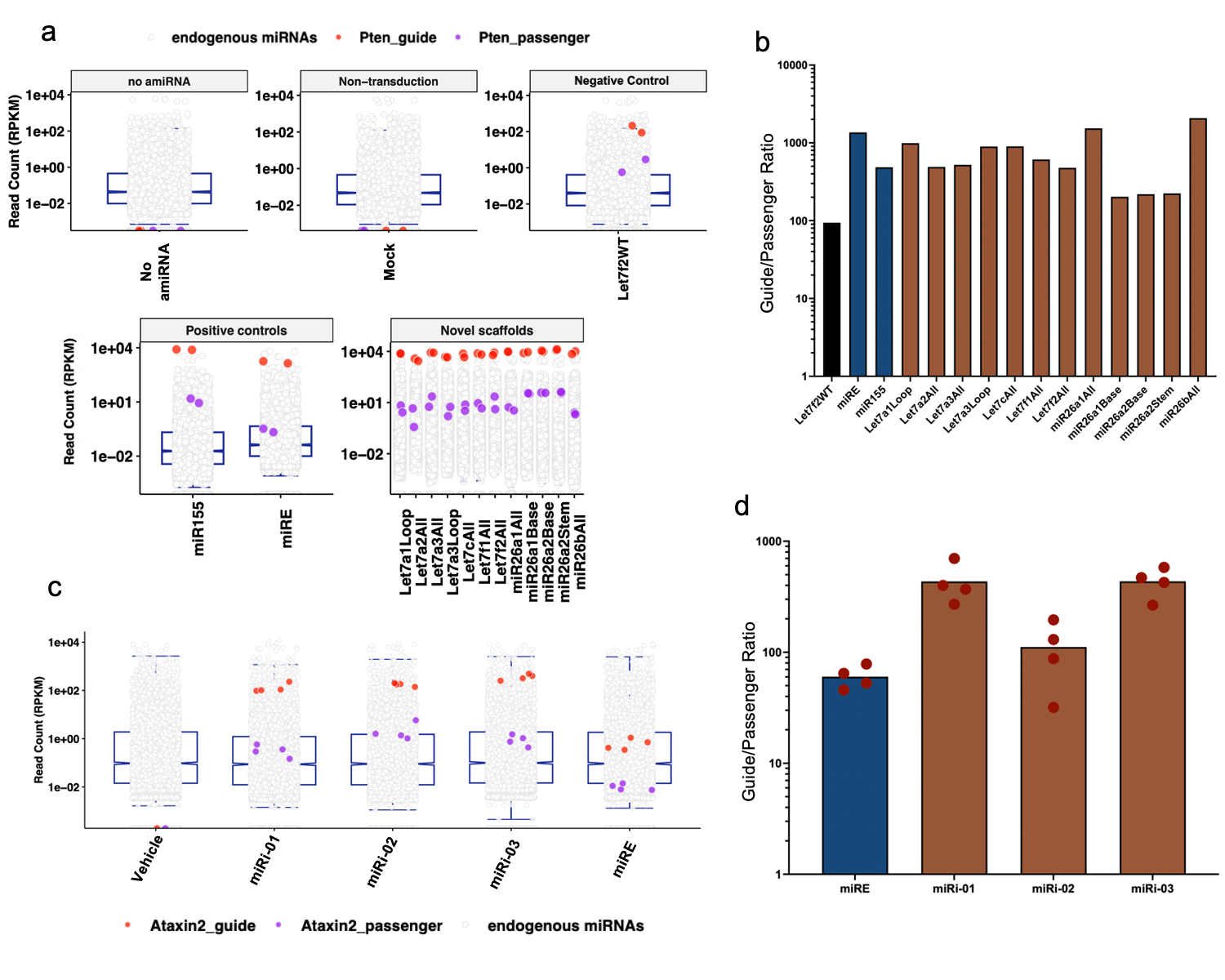

Supplement: Supplementary file 6 — Supplementary Material 6 [file 41598_2025_7061_MOESM6_ESM.tiff]

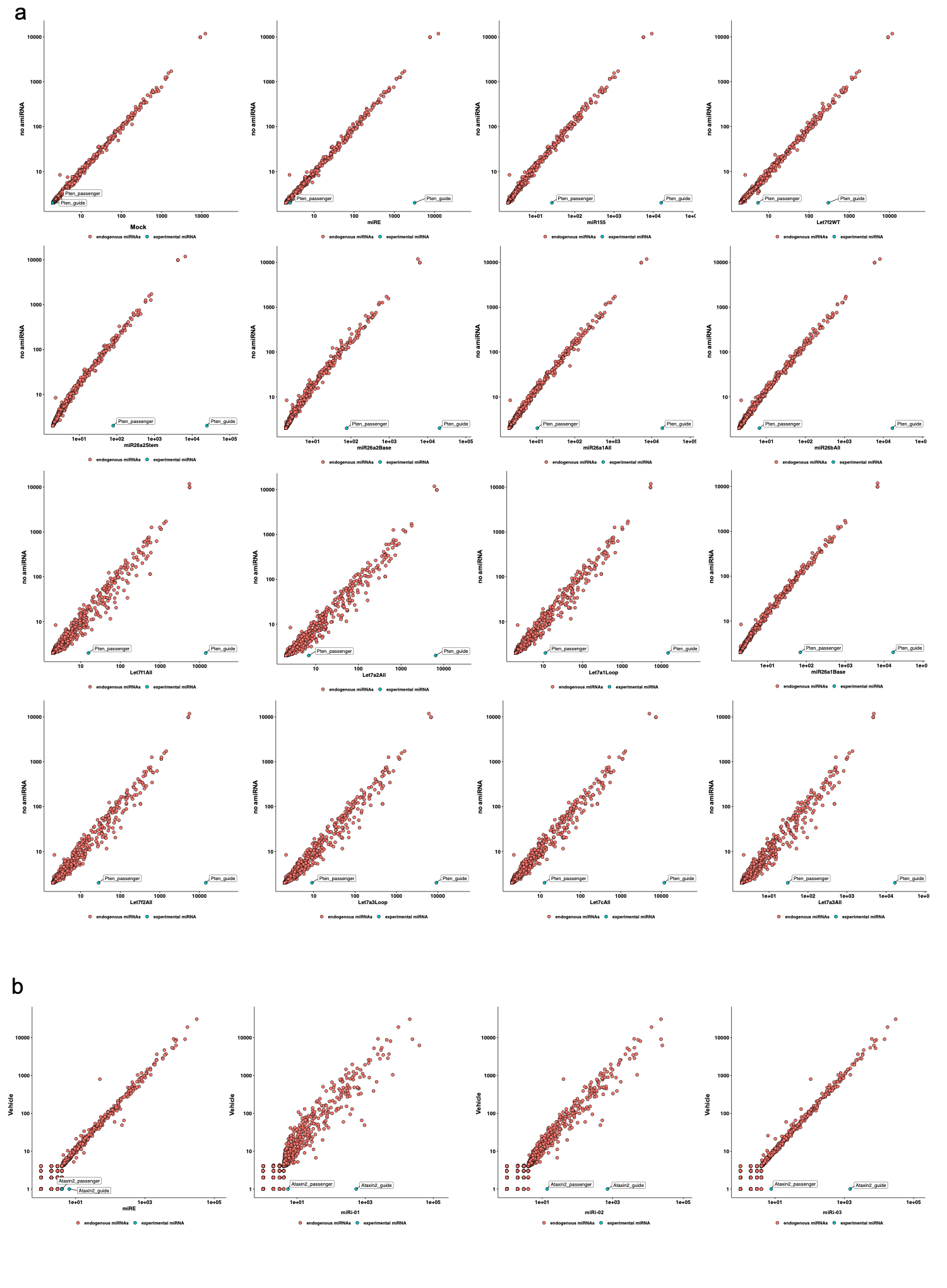

Supplement: Supplementary file 7 — Supplementary Material 7 [file 41598_2025_7061_MOESM7_ESM.tiff]

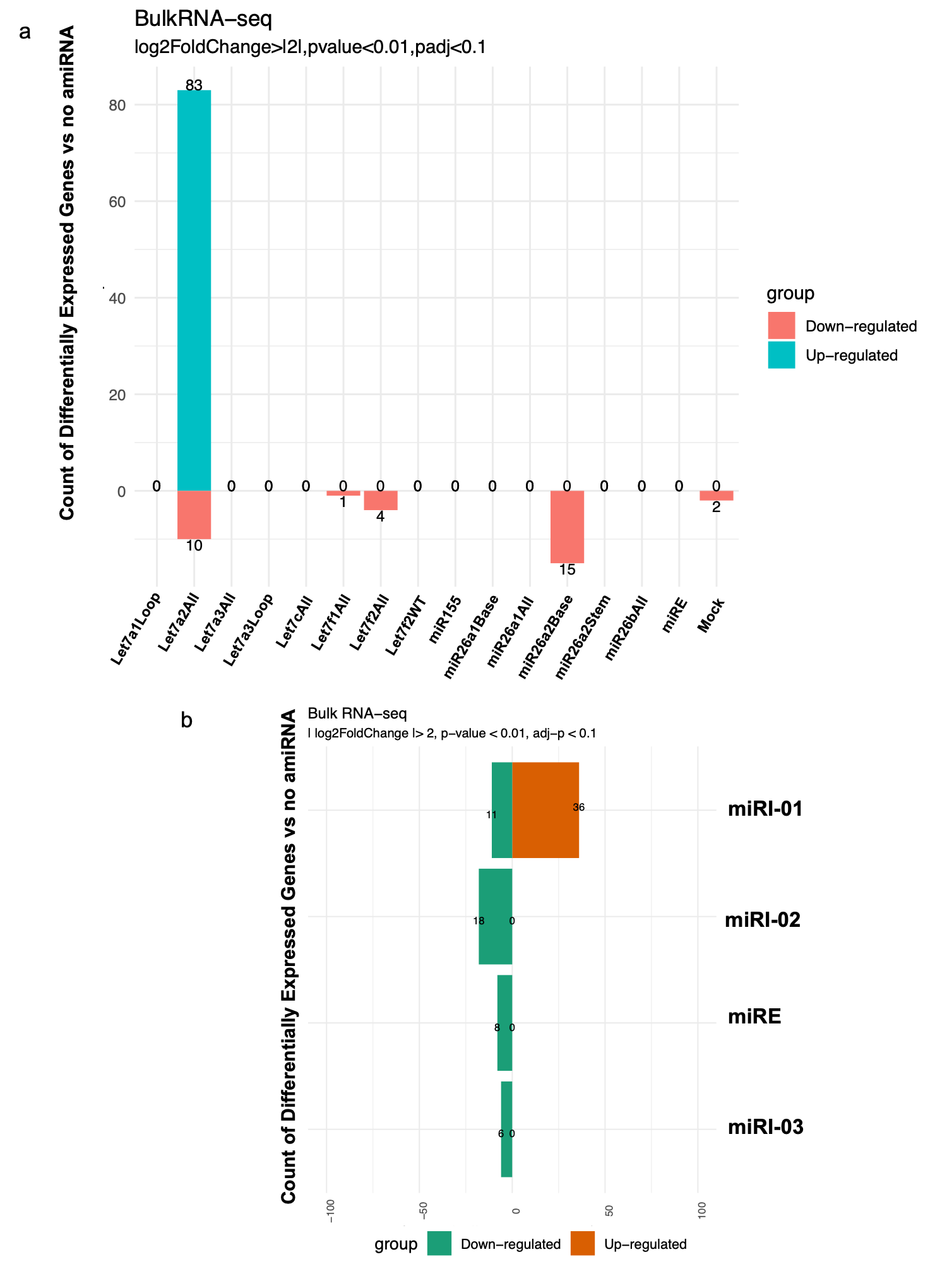

Supplement: Supplementary file 8 — Supplementary Material 8 [file 41598_2025_7061_MOESM8_ESM.tiff]

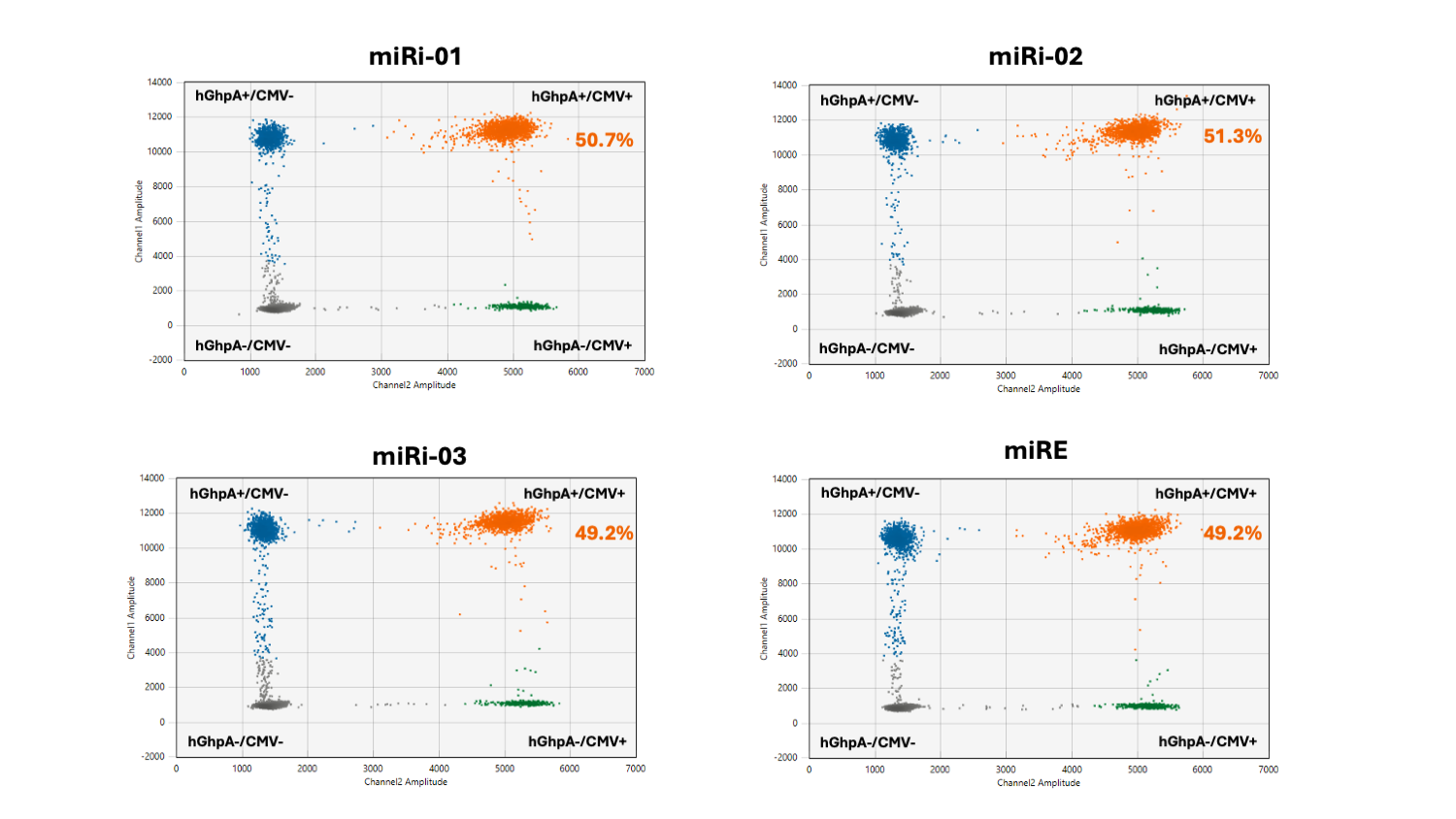

Supplement: Supplementary file 9 — Supplementary Material 9 [file 41598_2025_7061_MOESM9_ESM.tiff]
