# Supplementary material for: Engineered microRNA scaffolds for potent gene silencing in vivo
Source: Sci Rep. 2025 Jul 1;15:21419. doi: 10.1038/s41598-025-07061-y (PMC12218229; doi:10.1038/s41598-025-07061-y)
Supplement: Supplementary file 10 — Supplementary Material 10 [file 41598_2025_7061_MOESM10_ESM.docx]

**Supplemental Figure 1| Abundance of endomiRs, mature amiRNAs, and guide/passenger ratios.** a) Dot plot showing the normalized abundance of endogenous miRNAs (white dots) and the abundance of the guide (red dots) and passenger (purple dots) in NGN2 neurons infected with rAAV9 encoding the novel amiRNAs with a guide strand targeting *PTEN.* b) Guide/Passenger ratio, quantified from small RNA seq data, showing the high bias toward the guide strand arising from the amiRNAs expressed from the rAAV9 in NGN2 Neurons. c) Dot plot showing the normalized abundance of endogenous miRNAs (white dots) and the abundance of the guide (red dots) and passenger (purple dots) in cortexes infected with rAAV9 encoding the novel amiRNAs with a guide strand targeting *Ataxin2*. d) Guide/Passenger ratio, quantified from small RNA seq data, showing the high bias toward the guide strand arising from the amiRNAs *in vivo*.

**Supplemental Figure 2| Impact of the expression of amiRNAs on the miRome.** Scatter plots comparing the normalized expression of all the endogenous miRNAs (red blots) both a) *in vitro* and b) *in vivo.* In the first case, the comparison is made against the “no amiRNA” control (NGN2 neurons infected with a virus encoding GFP only). In the latter, the comparison is made against the “vehicle” control (mice cortexes injected with vehicle only). The quantification of the guide and passenger strands being produced from the amiRNAs is shown (blue dots).

**Supplemental Figure 3| Transcriptome analysis.** a) Bar graph showing the number of differentially expressed (DE) genes vs the “no amiRNA” control (NGN2 neurons infected with a virus expressing GFP only); two independent biological replicates per group were analyzed. b) Bar graph showing the number of differentially expressed genes vs the “vehicle” control (mice cortexes injected with vehicle only); four independent biological replicates per group were analyzed. P-values were calculated using one-way ANOVA while the adjusted p-values were calculated using the Benjamini-Hochberg false discovery rate (FDR) correction.

**Supplemental Figure 4 | 2D-ddPCR Quantification of rAAV9 Genome Integrity.** Data demonstrates uniform genome integrity profiles across all viral preparations, confirming consistent vector quality.
